# The structure of PghL hydrolase bound to its substrate poly‐γ‐glutamate

**DOI:** 10.1111/febs.14688

**Published:** 2018-11-19

**Authors:** Sneha Ramaswamy, Masooma Rasheed, Carlo F. Morelli, Cinzia Calvio, Brian J. Sutton, Annalisa Pastore

**Affiliations:** ^1^ The Randall Centre for Cell & Molecular Biophysics King's College London UK; ^2^ The Wohl Institute King's College London UK; ^3^ UK Dementia Research Institute at King's College London UK; ^4^ Department of Chemistry University of Milano Italy; ^5^ Department of Biology and Biotechnology University of Pavia Italy; ^6^ Department of Molecular Medicine University of Pavia Italy

**Keywords:** antimicrobial drug, biofilm inhibitor, PGA‐hydrolase, poly‐γ‐glutamate, virulence

## Abstract

The identification of new strategies to fight bacterial infections in view of the spread of multiple resistance to antibiotics has become mandatory. It has been demonstrated that several bacteria develop poly‐γ‐glutamic acid (γ‐PGA) capsules as a protection from external insults and/or host defence systems. Among the pathogens that shield themselves in these capsules are *Bacillus anthracis, Francisella tularensis* and several *Staphylococcus* strains. These are important pathogens with a profound influence on human health. The recently characterised γ‐PGA hydrolases, which can dismantle the γ‐PGA‐capsules, are an attractive new direction that can offer real hope for the development of alternatives to antibiotics, particularly in cases of multidrug resistant bacteria. We have characterised in detail the cleaving mechanism and stereospecificity of the enzyme PghL (previously named YndL) from *Bacillus subtilis* encoded by a gene of phagic origin and dramatically efficient in degrading the long polymeric chains of γ‐PGA. We used X‐ray crystallography to solve the three‐dimensional structures of the enzyme in its zinc‐free, zinc‐bound and complexed forms. The protein crystallised with a γ‐PGA hexapeptide substrate and thus reveals details of the interaction which could explain the stereospecificity observed and give hints on the catalytic mechanism of this class of hydrolytic enzymes.

**Abbreviations**
PDB: Protein Data Bank
rpm: revolutions per minute
γ‐PGA: poly‐γ‐glutamic acid

## Introduction

Poly‐γ‐glutamic acid (γ‐PGA) is a natural polymer composed by thousands of glutamates joined by γ‐peptide linkages. This type of bond, classically found in glutathione, connects the side chain carboxyl group in position γ of a Glu with the α amino group of the next residue. The unusual linkage prevents recognition and hydrolysis by classical proteases, which can only cleave α peptide linkages, allowing the polymer to survive proteolysis. Some bacterial species have the necessary biosynthetic machinery to perform the polymerisation reaction and secrete long chains of γ‐PGA. In microbial life, the functions exerted by γ‐PGA are mainly related to defence: γ‐PGA has a high water absorption capacity and the ability to chelate cationic compounds, including several toxic metals, and thus protects soil microorganisms both from desiccation and poisoning. Secretion of γ‐PGA into the cell surroundings also creates a physical barrier that prevents phage infections by shielding host receptors from viral recognition 1. Genes for γ‐PGA biosynthesis are abundantly present in soil microorganisms, particularly, but not exclusively, of the genera *Streptomyces*,* Staphylococcus* and *Bacillus*. However, a much larger number of organisms, including archaea and a few eukaryotes, produce and exploit the polymer 2.

γ‐PGA was first discovered as a component of the *B. anthracis* capsule. The capability of linking polymer chains on the outer cell surface allows the formation of a γ‐PGA capsule that protects bacteria from host immune surveillance, resulting in a significant impact on human health 3. The role of the γ‐PGA capsule as a fundamental virulence factor has been extensively established both for the Gram‐positive *B. anthracis* and for the Gram‐negative *Francisella tularensis,* both representing major biological threats 4, 5, 6, 7, 8. *Staphylococcus epidermidis* also uses γ‐PGA to evade host defences and its presence might be linked to persistence of some infections 9. The majority of γ‐PGA‐producing bacteria, including *S. epidermidis*, secrete a heterochiral polymer composed of D‐ and L‐Glu isomers (γ‐DL‐PGA) that surrounds bacteria without covalent attachment 1, 9. In contrast, the pathogen *B. anthracis* synthesises a 100% D‐Glu polymer (γ‐D‐PGA) covalently anchored to the peptidoglycan layer by the unusual γ‐glutamyltranspeptidase CapD 10. No information is available on the stereo composition of *F. tularensis* polymer. The lack of data depends on the inability to isolate γ‐PGA from this organism 11, possibly linked to its intracellular expression in host macrophages, where γ‐PGA appears to play a role in *F. tularensis* phagosomal escape and/or arrest of phagosomal maturation 7.

Much government defence research is aimed at preventing or destroying the γ‐PGA capsule 12, 13, 14. This aim is thwarted by the polymer structure itself, which confers resistance to common proteases. γ‐PGA degradation requires specific enzymes 15. Among those currently known, three classes can be distinguished. The first class includes enzymes belonging to the γ‐glutamyl transferase (GGT) family (EC2.3.2.2; T03.001 in MEROPS peptidase database 16), which hydrolyse γ‐PGA from its amino terminal end in an exotype manner with no stereospecificity 15. CapD is a GGT‐like enzyme present in *B. anthracis* genome (T03.023 in MEROPS) that normally acts by severing the growing γ‐PGA chain from the biosynthetic machinery and linking it to the bacterial surface. Enzymatic degradation of the γ‐PGA capsule of *B. anthracis* with high concentrations of purified CapD enhances phagocytosis and killing of bacteria by neutrophils 12, 13, 14. A second class of γ‐PGA‐degrading enzymes is represented by *B. subtilis* PgdS (poly‐glutamate degradation), a member of the CHAP (cysteine, histidine‐dependent amidohydrolases/peptidases) superfamily 17 (C40.005 in MEROPS). The recombinant enzyme hydrolyses γ‐PGA into large L‐glutamate‐rich (200–450 kDa) fragments and D‐glutamate‐rich small oligopeptides (2–5 kDa), probably acting between two D‐Glu residues 18, 19, 20. The third class of enzymes is typified by PghP (poly‐γ‐glutamate hydrolase of phage; M86.001 in MEROPS), a zinc‐binding enzyme identified in a *B. subtilis* natto phage 21. These enzymes are extraordinarily effective in efficiently degrading the polymer into small oligomers but are only able to target γ‐DL‐PGA, while they are ineffective against the *B. anthracis* capsule 22. This specificity has been suggested to be due to the stereocomposition of *B. anthracis* γ‐PGA.

Recently, four unannotated *B*. *subtilis* gene products, YjqB, YmaC, YndL, and YoqZ, were found to share with PghP high sequence similarity (27–37% identity and 41–54% homology) 2. The authors also demonstrated that recombinant YndL and YoqZ were efficient at γ‐DL‐PGA degradation thus highlighting their functional homology to PghP. These proteins were thus renamed by homology PghB, PghC, PghL and PghZ respectively. Their genes are likely derived from integrated prophages, as judged by their localisation in prophagic regions of the *B. subtilis* genome 2.

Understanding how γ‐PGA‐degrading enzymes work is an important goal. In *B. anthracis, F. tularensis* and *S. epidermidis,* enzymatic degradation of the γ‐PGA capsule or lack of γ‐PGA synthesis has been shown to drastically mitigate bacterial virulence in animal models, allowing infected organisms to develop appropriate immune responses by neutrophils 5, 9, 12, 13, 14. In the long term, such results will contribute to the development of a therapeutic derivative of PghP‐like enzymes as a tool against persistent infections caused by γ‐PGA‐producing pathogenic bacteria.

With the aim of understanding the activity and stereoselectivity of γ‐PGA hydrolases ultimately aimed at exploring their potential use as therapeutics for the treatment of recalcitrant infections, we have solved the structure of the recombinant PghL hydrolase from *B. subtilis*. We had previously demonstrated that the recombinant protein is fully enzymatically active and able to efficiently cut γ‐PGA 2. We now characterised further the cleavage properties of this enzyme and crystallised PghL both in isolation and in the presence of γ‐PGA and solved the structures of a zinc‐free, zinc‐loaded and γ‐PGA‐enzyme complex. Our results offer high resolution details of the interaction of PghL with the substrate and provide insights into the basis for its specificity.

## Results

### Characterisation of PghL stability

**Figure 1:**
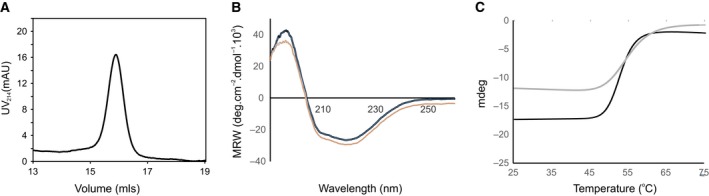
PghL is a folded and relatively stable protein. (A) Size exclusion chromatography profile of WT‐PghL. The concentration of the protein used was 0.05 mg·mL ^−1^. (B) Comparison of the far‐UV spectrum of apo (grey) and native PghL (black) at 30 °C. (C) Thermal scan of PghL monitored by CD. Black curve: PghL in 20 mm sodium phosphate, at pH 6.0 using a protein concentration of 5 μm. The calculated melting point was 55 °C. Grey curve: PghL in 5 mm Hepes and 150 mm NaF at pH 6.0 using a protein concentration of 5 μm. The calculated melting point was 53 °C.

We first characterised PghL for its folding and stability. The protein is monomeric at room temperature as judged from analytical size exclusion chromatography (Fig. 1A). CD confirmed that the protein is stably folded. The spectrum is typical of an α‐helical rich structure with minima at 208 and 222 nm (Fig. 1B). The protein has an apparent temperature of unfolding in Hepes and phosphate buffers of 53 °C and 55 °C respectively (Fig. 1C). However, the reaction is irreversible indicating aggregation, as also suggested by the higher content of a β‐rich structure after heating (data not shown). Since the PghP homologue is a zinc‐bound metallopeptidase, we expected that PghL could contain a metal ion. The native protein (as solubly expressed and purified) was thus treated with EDTA. The CD spectra and the stabilities of the EDTA‐treated and untreated proteins were very similar with melting points in phosphate of 53 °C and 55 °C respectively, suggesting that, if present, zinc is not structurally important.

### Characterisation of the enzymatic activity of PghL

**Figure 2:**
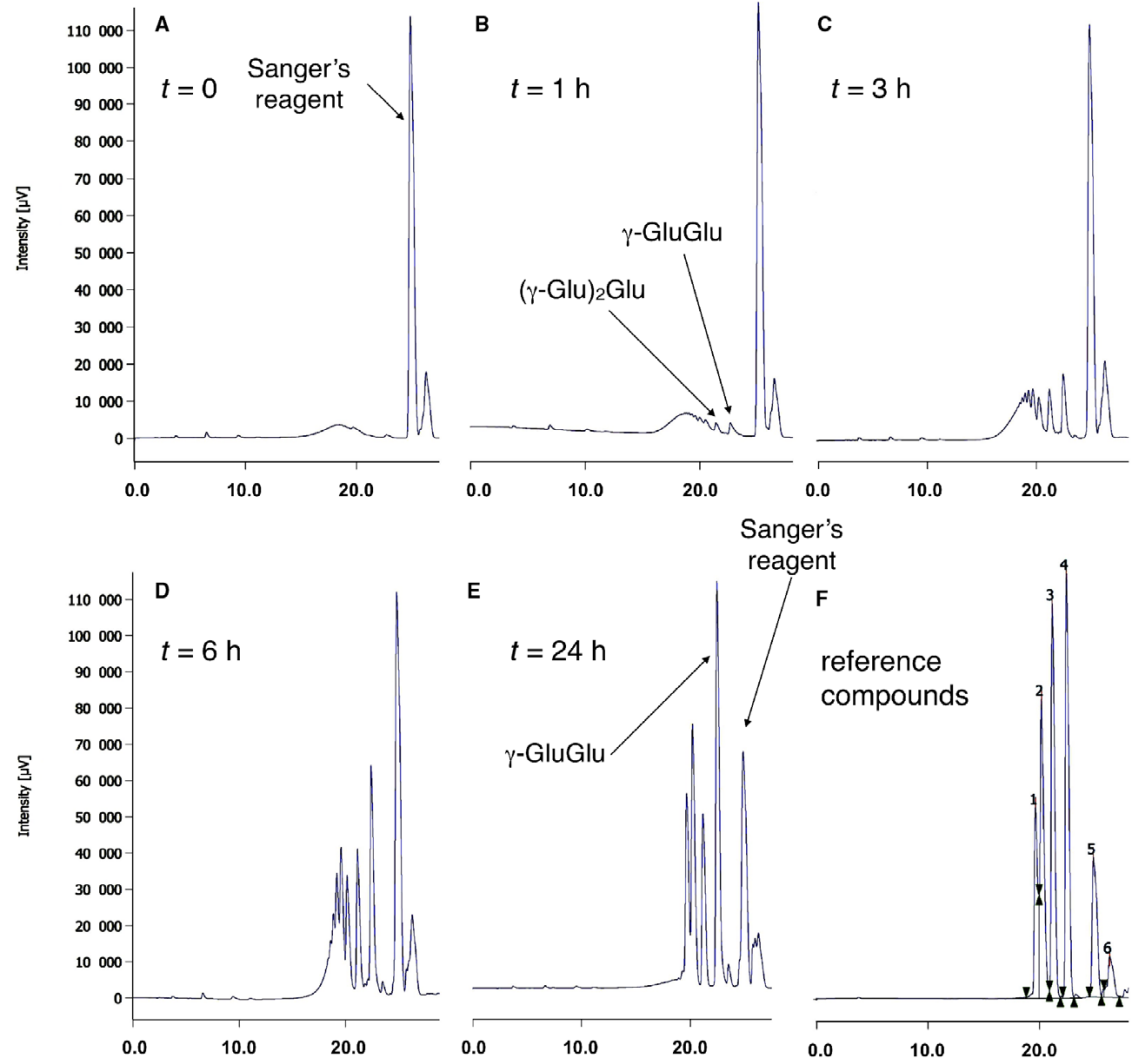
PghL‐catalysed hydrolysis of γ‐PGA. Representative chromatograms showing the appearance of reaction products over time. HPLC chromatograms for: (A) Time point 0: only the Sanger reagent is present. (B) After 1 h: small peaks with the retention times of dimers (γ‐GluGlu) and trimers (γ‐Glu‐γ‐GluGlu) appear. (C) After 3 h: peaks attributable to dimers and trimers are well defined; other peaks attributable to higher molecular weight oligomers emerge from a broad peak caused by the progressively degraded polymeric material. (D) After 6 h: peaks attributable to up to hexamers are clearly visible. Signals of putative hepta‐ and octapeptide are distinguishable as shoulder peaks. (E) After 24 h: only peaks assigned to the di‐, tri‐, tetra‐ and pentapeptide are visible. (F) chromatogram of a mixture of authentic di‐ (4), tri‐(3), tetra‐(2) and penta‐peptides (1) used as reference compounds is resolved. Peak 5 is due to the excess Sanger's reagent used for pre‐column derivatisation. Peak 6 accompanies peak 5 as an unidentified impurity.

We characterised the γ‐PGA hydrolysis products and the enzyme stereoselectivity. To determine the products released by PghL, the reaction was monitored by HPLC upon pre‐column derivatisation. During the course of the reaction, low‐molecular weight γ‐PGA fragments composed of two or more glutamic acid residues were initially observed (Fig. 2A). Longer products appeared gradually from the indistinct envelop of the high‐molecular weight species and were progressively reduced to short oligomers (γ‐GluGlu and γ‐Glu‐γ‐GluGlu; Fig. 2B–E). Peaks attributable to longer oligomers (up to eight glutamic acid residues) remained distinguishable in the chromatograms within the first 6 h (Fig. 2D). At 24 h, only oligomers composed by 2–5 glutamic acid residues were clearly distinguishable from the higher molecular weight background (Fig. 2E). The pattern did not change upon prolonged incubation (data not shown). Free glutamic acid was not detected in the reaction mixtures, thus confirming that PghL is an endo‐hydrolase 21. Using higher amount of enzyme, only γ‐GluGlu dimer and γ‐Glu‐γ‐GluGlu trimer accumulated after 24 h (data not shown).

**Figure 3:**
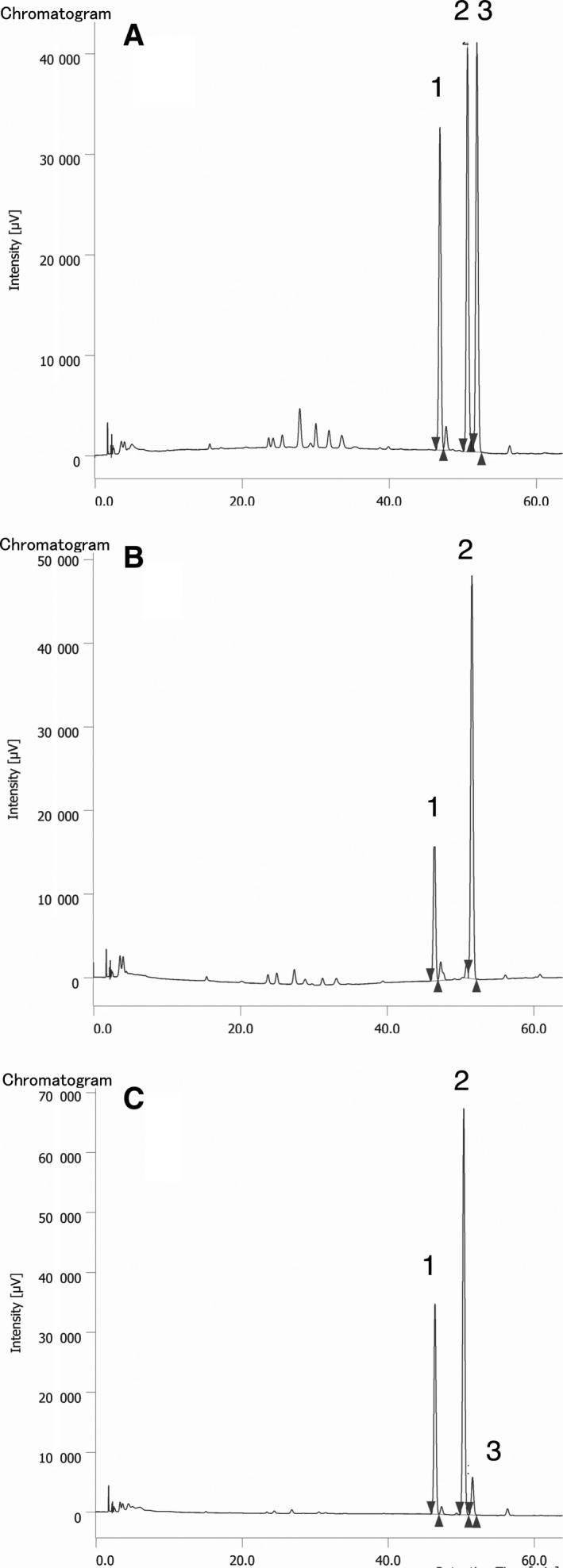
Stereochemical outcome of PghL‐catalysed hydrolysis of γ‐PGA. Chromatograms were obtained after acidic hydrolysis of the samples and pre‐column derivatisation of the resulting glutamic acid with Nα‐(2,4‐dinitro‐5‐fluorophenyl)‐L‐valinamide. (A) Starting γ‐PGA after acidic hydrolysis; 1 = L‐Glu, 2 =  D‐Glu, 3: Nα‐(2,4‐dinitro‐5‐fluorophenyl)‐L‐valinamide. (B) γ‐GluGlu from enzymatic reaction after acidic hydrolysis; 1 = L‐Glu, 2 = Nα‐(2,4‐dinitro‐5‐fluorophenyl)‐L‐valinamide. (C) High‐molecular weight fraction from PghL‐catalysed reaction after acidic hydrolysis; 1 = L‐Glu, 2 =  D‐Glu, 3: Nα‐(2,4‐dinitro‐5‐fluorophenyl)‐L‐valinamide.

The stereospecificity of PghL was established upon isolation of both the low‐molecular weight fragments and the high‐molecular weight species produced by enzymatic digestion. γ‐GluGlu and γ‐Glu‐γ‐GluGlu were individually separated by ion exchange chromatography and verified by ^1^H NMR spectroscopy, while the higher molecular weight fraction was recovered by dialysis against water using a membrane with a cut‐off of 3,500 Da. After acidic hydrolysis of the original γ‐PGA substrate and of each individual fraction, the free glutamic acid released was derivatised with the chiral Nα‐(2,4‐dinitro‐5‐fluorophenyl)‐L‐valinamide and analysed by HPLC. While the starting material contained D‐ and L‐ glutamic acid residues in a ca. 54 : 46 ratio (Fig. 3A), the γ‐GluGlu and the γ‐Glu‐γ‐GluGlu oligomeric fractions exclusively contained glutamic acid in L‐configuration (Fig. 3B). Only in the higher molecular weight fraction, D‐glutamic acid could be detected, as its ratio rose with respect to the L‐enantiomer (66D:34L; Fig. 3C). The absence of D‐glutamic acid in the oligomeric fractions and its accumulation in the residual material demonstrate the stereospecificity of PghL for γ‐PGA chains containing L‐glutamic acid residues only.

### The enzyme structure

**Figure 4:**
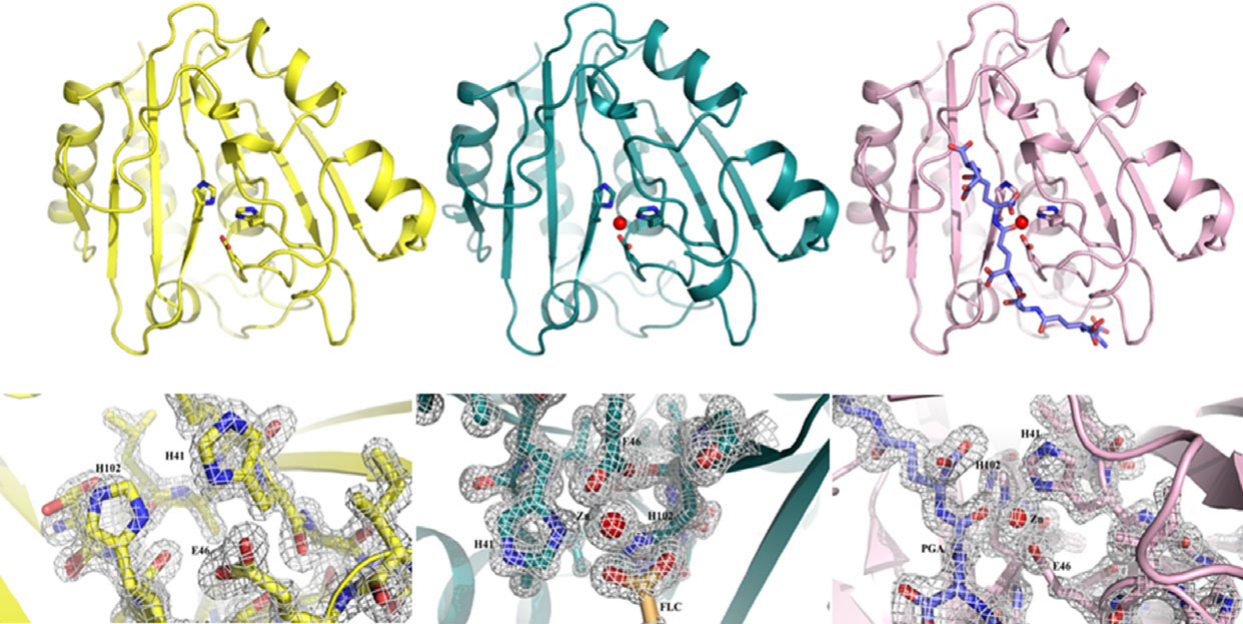
PghL crystal structures. Top panel: Ribbon diagrams for apoPghL (in yellow, left), native PghL (in deep teal, middle) and PghL‐γ‐PGA complex structure (in light pink, right; γ‐PGA shown in blue). Residues involved in zinc coordination are shown as sticks. Bottom panel: 2Fo‐Fc maps for apoPghL with the protein in yellow (left),native PghL with the protein in deep teal and sodium citrate (FLC) in light orange (middle)and PghL‐γ‐PGA in light pink and γ‐PGA in blue (right). The maps were contoured at 2σ.

The crystal structures of native, zinc‐free (apo) and a γ‐PGA‐complexed PghL were determined by molecular replacement at resolutions of 1.03, 1.7 and 1.7 Å, respectively, using the crystal structure of the PghP homologue Protein Data Bank (PDB accession code: 3a9l; Fig. 4). (Note that the completeness in the outer shell of the native structure is not as high as the others (Table 1)). The asymmetric units for all three structures contain one PghL molecule hydrated by water. The native protein and the complex also contain one zinc ion, and sulphate ions are observed in the apo and native structures. PghL is a globular protein with a α/β structure: a seven‐stranded β‐sheet is arranged according to a β1, β3, β2, β4, β7, β5, β6 topology, interleaved by six α‐helices and five short 3_10_ helices. The helices protect the hydrophobic surface of the core β‐sheet, while the hydrophilic surface regions of the sheet are exposed to the solvent. The Zn^2+^ ion is located near the ends of strands β2 and β4. The loops between β3 to α3 and β6 to β7 together with the core β‐sheet form a cleft. In the structure of apo PghL, the residues involved in Zn^2+^ coordination are in the same conformation as in the zinc‐bound (native) structure.

**Table 1:** X‐ray data collection and refinement statistics

	Apo PghL	Native PghL	PghL‐PGA complex
Data statistics			
Space group	P2_1_2_1_2_1_	P2_1_2_1_2_1_	P2_1_2_1_2_1_
Number of molecules/asymmetric unit	1	1	1
Cell dimensions (Å)	*a *=* *38.7	*a *=* *38.7	*a *=* *38.6
	*b *=* *47.3	*b *=* *46.8	*b *=* *47.7
	*c *=* *99.9	*c *=* *100.07	*c *=* *99.3
Resolution range (outer shell), (Å)	15.75–1.7 (1.76–1.7)	34.2–1.03 (1.07–1.03)	15.74–1.7 (1.76–1.7)
*R*_symm_ (outer shell), (%)	4.0 (21.7)	2.2 (37.6)	2.4 (13.9)
I/σI (outer shell)	22.7(3.15)	18.67 (1.34)	24.58 (3.98)
Completeness (outer shell), (%)	99 (96)	85 (21)	99 (96)
Multiplicity (outer shell)	2.0 (1.9)	1.9 (1.2)	2.0 (1.9)
Total number of reflections (outer shell)	40 627 (3713)	14 679 (2333)	40 956 (3757)
No. of unique reflections (outer shell)	20 566 (1942)	76 651 (1872)	20 613 (1933)
Wilson B‐factor (Å^2^)	17.25	11.71	14.34
CC(1/2)	0.79 (0.35)	0.99(0.63)	0.99 (0.91)
Refinement statistics			
*R*_cryst_ (%)	17.7	17.3	15.7
*R*_free_ (%)	21.2	18.9	18.9
Number of non‐H atoms	1867	1916	1902
Protein	1629	1636	1618
Ligand	36	42	56
Water molecules	213	238	255
Other molecules	4	4	2
Average B‐factor (Å^2^)			
Overall	19.85	15.21	16.92
Protein	18.32	13.61	15.35
Ligand	35.74	19.79	20.87
Solvent	29.32	24.65	27.08
RMSD in			
Bond length (Å)	0.011	0.009	0.006
Bond angle (°)	1.67	1.51	0.79
Ramachandran statistics			
Favoured (%)	98	98	99
Outliers (%)	0	0	0
RCSB PDB Code	6hrj	6hri	5onj

The three structures have a similar fold, with rmsd between the backbone atoms of 0.15–0.19 Å. This confirms that zinc does not modify the structure, in agreement with the similar thermal unfolding behaviour of the apo and native proteins. Minimal structural differences are observed for residues 142–146, which are located in the loop between strands β6 and β7 and positioned at the edge of the catalytic cleft. Electron density for residues 7–207 are observed for all three structures. In the apo and in the complex structures, there is an additional N‐terminal alanine residue visible; the last seven residues of the LEHHHHHH C‐terminal tag inserted for purification purposes are not observed in any of the structures. The side chain of the solvent exposed Arg190 is observed in two different conformations one of which appears to be stabilised by π stacking interactions with the adjacent Phe133.

### Zinc coordination and comparison with classical carboxypeptidases

**Figure 5:**
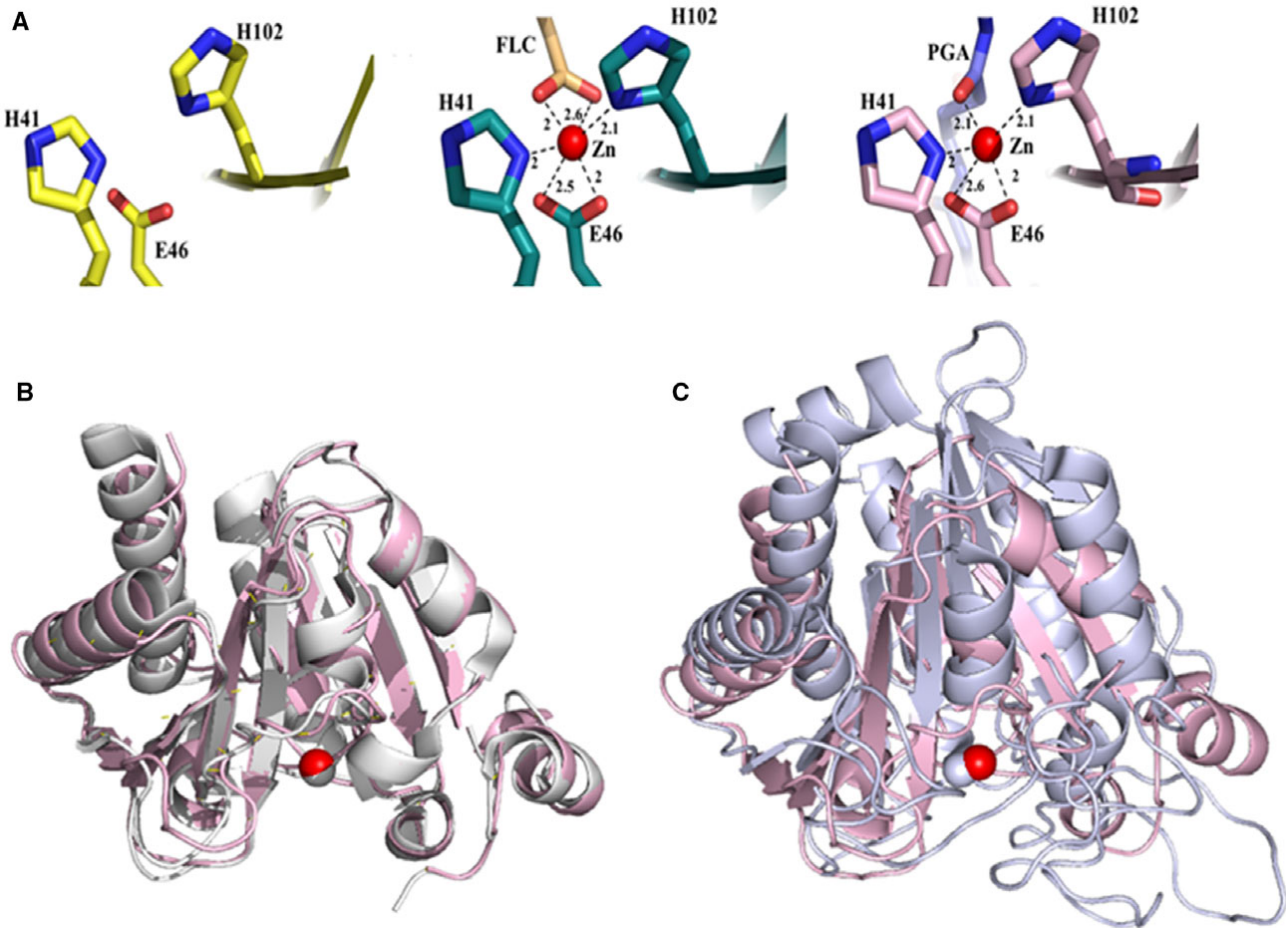
Zinc coordination site and comparison of PghL with carboxypeptidases. (A) Residues involved are shown as sticks. Apo PghL (left, in yellow), native PghL (middle, in deep teal; FLC: sodium citrate) and PghL‐γ‐PGA complex structure (right, in light pink; γ‐PGA shown in blue). Distances are in Å. (B) Superposition of PghL (in pink) and PghP (PDB code: 3a9l, in white). (C) Superposition of PghL (in pink) and carboxypeptidase A (PDB code: 3cpa, in grey) as superposed by the Dali server (http://ekhidna.biocenter.helsinki.fi/dali_server/). Zinc atoms are shown as grey or red spheres.

In both the structures of native and γ‐PGA‐complexed PghL, coordination of the Zn^2+^ ion has contributions from the side chain Nδ1 atoms of His41 and His102, and the two carboxyl oxygens of Glu46 in a bidentate mode. In native PghL, hexa‐coordination results from the further contribution of sodium citrate from the crystallisation solution, while in the complex, penta‐coordination occurs with the carbonyl oxygen atom of Glu4 in the bound γ‐PGA hexapeptide (Fig. 5A). Thus, although different, the two small molecules both participate in zinc binding in a topologically equivalent way. His77 and His144 lie close by, but the former is too far away to participate in coordination, and the side chain of the latter points away from the zinc ion. No ordered water molecule is sufficiently close to the zinc or to the nearby side chains.

Comparison of the coordinates with the entire PDB using the Dali server (http://ekhidna.biocenter.helsinki.fi/dali_server/) resulted in a large number of hits which include mostly carboxypeptidases. The higher hits are mainly bacterial proteins among which the close homologue PghP from phage ΦNIT1 is of course the closest structure (3a9l, *Z* score 32.3 and rmsd 1.2 Å on 188 residues according to Dalilite; Fig. 5B). After this, the following hits identified a hypothetical protein from *Agrobacterium tumefaciens* (2odf, *Z* score 12.7 and rmsd 2.9–3.1 Å depending on the chain) and an *N*‐formylglutamateamido‐hydrolase from *Ralstonia eutropha* (2q7s, *Z* score 12.2 and rmsd 3.1 Å). Bovine carboxypeptidase A (PDB accession code: 3cpa), a protein well characterised and often used as a reference for this enzyme family, also appears but with a much lower score (*Z* score 8.9 and rmsd 3.2 Å; Fig. 5C).

The residues which coordinate the zinc ion are strongly conserved between the structures of PghP and other non‐γ‐PGA‐specific carboxypeptidases such as carboxypeptidase A, if not in their sequential position, then in their three‐dimensional location. PghL residues His41 and His102, and the two carboxyl oxygens of Glu46 in a bidentate mode overlap well, for instance, with His40, His103 and Glu45 of PghP and are conserved in other homologues 2. The zinc atom sits precisely in the same position as observed in PghP with which it also shares a similar environment (Fig. 5B). The involvement of these conserved residues in catalysis is testified by the fact that mutations of His40, Glu45, His78, His103 and Glu165 to alanine in PghP (equivalent to His41, Glu46, His77, His102 and Glu165 in PghL) have been reported to have a deleterious effect upon γ‐PGA‐degrading activity 22. Glu165 of PghL at the C terminus of β7 is also spatially equivalent to Glu270 in carboxypeptidase A, although the degree of sequence and structural homology is much lower.

### The structure of a γ‐PGA complex

**Figure 6:**
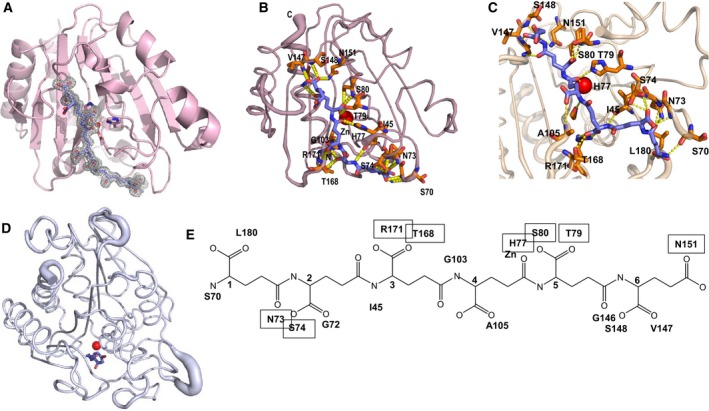
The structure of a PghL‐γ‐PGA complex. (A) Omit map contoured at 1σ showing the electron density for the hexapeptide; PghL is shown in ribbon representation (light pink). (B) PghL‐γ‐PGA complex structure with the enzyme in light pink and γ‐PGA shown in blue. Residues involved in hexapeptide coordination are shown as sticks. The zinc ion is shown in red. (C) Close‐up and network of interactions. The view is approximately rotated by 120° as compared to (B). (D) Carboxypeptidase A (3cpa; shown in grey) interacting with a tyrosine residue that mimics the substrate represented in the same view as PghL. The two structures were first structurally superposed. The zinc ion is shown in red. (E) Formula of the hexapeptide indicating the interactions with the enzyme and the zinc ion. Interactions that involve the protein side chains, and are thus sequence‐specific, are boxed. The alpha carbons of PGA are labelled sequentially.

Electron density for a hexameric γ‐PGA peptide was observed in the structure obtained from the crystals grown in 0.1 m sodium acetate, pH 5.0, 5% w/v γ‐PGA and 20% polyethylene glycol 3350 and crystallised using screens (from Molecular Dimensions) which contain γ‐PGA as a new strategy of crystallisation. This method exploits the high nucleation‐precipitation potential of the polymer (average molecular weight 200–400 kDa) which enables its use at very low concentrations in combination with classical precipitants. In the structure, there is clearly defined electron density for a bound hexapeptide, which must have been trapped from the crystallisation medium (Fig. 6A). Since it is highly unlikely that the high‐molecular weight Molecular Dimensions material (200–400 kDa) contained sufficient quantities of a hexapeptide, we presume that γ‐PGA from the medium must have been hydrolysed during crystallisation which trapped the hexapeptide. This binds to the catalytic site and, head‐to‐tail, approximately spans the whole length of the protein and packs against it (Fig. 6B). Any additional residue of γ‐PGA would overhang the protein and be highly exposed to the solvent.

All six residues of the peptide are in an L‐configuration. The γ‐PGA fragment is positioned in such a way that the side chain carboxyl group of Glu4 in γ‐PGA is in close proximity to the zinc (distance 2.1 Å; Fig. 6B,C), indicating that this atom plays a functional role in the hydrolysis of γ‐PGA by PghL, as expected for zinc carboxypeptidases. This tight anchoring could explain the specificity of this family of enzymes for γ‐PGA rather than for any other polypeptide chain: in classical carboxypeptidases, the main anchoring occurs through interactions between enzyme residues (Arg145 and Tyr248 in carboxypeptidase A) with the amides of the scissile site (position i) and position i + 1. The interactions observed in PghL involve instead residues all throughout the peptide substrate including groups at positions i‐3, i‐2, i‐1, i + 1 and i + 2. This allows anchoring to a homopolymer chain with a very unusual spacing: the NH‐NH distances in an extended γ‐PGA chain are typically around 6.2 ± 0.3 Å, compared to the distance in an extended conventional polypeptide chain of around 3.8 Å. Intriguingly, the position of Glu5 in the γ‐PGA peptide is roughly equivalent to that of the smaller dipeptide (Gly‐Tyr) observed in the structure of bovine carboxypeptidase A (3cpa) in which the tyrosine was suggested to mimic the substrate (Fig. 6D).

**Figure 7:**
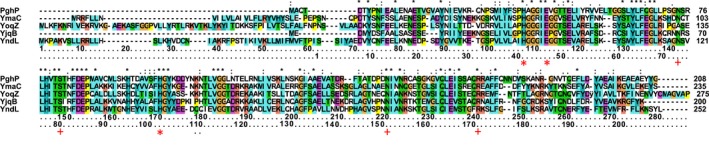
Sequence alignment of the γ‐PGA cleaving proteins from *B. subtilis*. The numbering of the PghL construct used in this study is indicated in the last row, whereas the numbering according to the UNIPROT entries are given in the row above. The residues involved in zinc binding and γ‐PGA sequence‐specific interactions are indicated with red stars and plus symbols respectively.

An extensive network of hydrogen bonds are formed between the enzyme side chains of Asn73, Ser74, His77, Thr79, Ser80, Asn151 and Thr168 and Arg171 and the backbone carbonyls of Ile45, Ser70, Gly72, Gly103, Ala105, Gly146, Val147, Ser148 and Leu180 with the carboxyl groups of the hexapeptide (Fig. 6C,E). These interactions are in excellent agreement with, and explain features observed in the structure of PghP, where the active site hosts a phosphate ion whose oxygen atoms form hydrogen bonds with the side chains of His78,Thr80, Ser81, Glu165 (equivalent to His77, Thr79, Ser80 and Glu165 in PghL) and the main chain nitrogen atom of Ser81. Mutagenesis of Thr80 of PghP into an alanine has been reported to result in almost complete abolishment of the activity of the wild‐type enzyme 22. These residues are not conserved in classical carboxypeptidases, where they are replaced by quite different conserved residues (Fig. 7). In carboxypeptidase A (3cpa), for instance, Arg127, Asn144 and Arg145 occupy the space roughly equivalent to His77, Thr79 and Ser80 of PghL (data not shown). The PghL residues are thus likely to be involved in substrate recognition and the key to provide substrate specificity for γ‐PGA rather than for conventional polypeptide chains.

**Figure 8:**
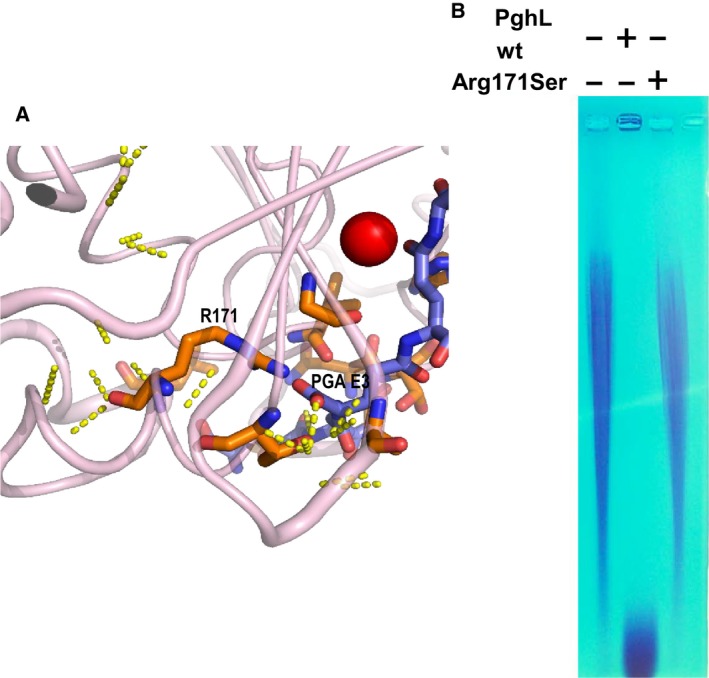
The key role of Arg171Ser in γ‐PGA anchoring. (A) Close‐up of the interaction between Arg171 and Glu3 of γ‐PGA. (B) Degradation by wild‐type and Arg171Ser mutant PghL. γ‐PGA from *B. subtilis* was treated with 100 ng of either wild‐type or Arg171Ser mutant hydrolase for 1 h at 37 °C and separated on a 2% agarose gel in TAE buffer. Identity of the enzyme is indicated above.

Among the residues interacting through the side chain a potentially important feature is the presence of Arg171 which firmly anchors the carboxyl group of the third γ‐PGA glutamate (Fig. 8A). Together with His77, Thr79 and Ser80, this residue conserved within *B. subtilis* γ‐PGA hydrolases could be involved in determining the stereospecificity of this enzyme for γ‐L‐PGA as compared to enzymes that cleave γ‐D‐PGA or γ‐LD‐PGA chains 2, 10. To test this hypothesis, we designed an Arg171Ser mutant and tested it for polymer degradation. According to our prediction, the Arg171Ser mutation completely abolished γ‐LD‐PGA cleavage (Fig. 8B).

Taken together, these results provide significant new insight into the specificity of γ‐PGA hydrolysis, the stereospecificity of PghL and provide an important guideline to the way cleavage occurs.

## Discussion

We have described here the structure of PghL, a member of the γ‐PGA‐specific carboxypeptidase family recently identified in several bacterial species 2. These enzymes are interesting for several reasons. From an evolutionary point of view, previous studies have demonstrated that *pghP*‐like genes originate from phages and spread to several bacterial through horizontal gene transfer 2.

Importantly for us, these robust, small and resistant enzymes hold potential to be engineered into therapeutic agents against recalcitrant bacterial infections sustained by γ‐PGA‐producing pathogens. Detailed structural knowledge is nevertheless the essential prerequisite to maximise the possibility of developing a new class of therapeutic agents that target specifically γ‐PGA chains with a particular stereochemical composition. If this objective was reached, the proposed treatment could expose bacteria to the innate host defence system without causing direct bacterial death and could thus be less likely to induce bacterial resistance.

The high‐resolution structures of PghL that we reported here provide insights into the catalytic mechanism both of carboxypeptidases in general and of PGA hydrolases in particular. Two models have been suggested for the first step of the catalytic hydrolysis by carboxypeptidases. The carboxylate side chain of the distal glutamate (Glu270 in human carboxypeptidase A) either directly attacks the scissile carbonyl carbon to form an acyl‐enzyme intermediate (anhydride mechanism) or acts indirectly through activation of a zinc‐coordinated water molecule to form a tetrahedral intermediate (water‐promoted mechanism). Despite numerous experimental studies, there is still no definitive evidence to favour one mechanism over the other. In the PghL structure, however, no crystallographically defined water molecule is sufficiently close to the zinc atom to participate in the reaction, suggesting a mechanism that is not water‐mediated. On the other hand, other interpretations are possible: the role of water in zinc coordination could be played by a citrate ion from the crystallisation buffer in the native structure, and by a carbonyl group of the bound gamma‐PGA hexapeptide in the complex. It is known that, at pH values below the pKa of the distal glutamate, the stabilisation of the zinc‐coordinating water is weakened and this ligand could thus easily be displaced by other ligands. We should also take into account that the bound hexapeptide is assumed to be a substrate, but the cleavage data reported here indicate that it is a primary cleavage product and a nonideal substrate. This could suggest that the hexapeptide is bound to the enzyme in an inhibitor‐like manner, displacing the catalytic water as is usual for inhibitors of this class of enzymes.

We tried to crystallise PghL using γ‐PGA directly purified from *B. subtilis* but did not succeed, whereas we readily obtained crystals using the Molecular Dimensions screens that contained γ‐PGA (200–400 kDa) under various conditions. All our crystals contained a hexapeptide that must have been generated by catalytic degradation of longer peptide chains, since it is highly unlikely that sufficient quantities of a hexapeptide would be present in the medium despite the polydispersity of the polymer. This suggests that perhaps the viscosity or other biophysical properties of the longer γ‐PGA peptides are unfavourable for crystallisation. Future studies will need to take into account this observation. Accordingly, we showed that PGA hydrolysis progresses through the formation of short oligomers down to the level of hexamers and pentamers, and can proceed, with slower kinetics, to form dimers and trimers. These results are fully compatible with the structural information: in the solved structure, a hexamer of γ‐PGA is bound to the active site of the enzyme. It is therefore reasonable to assume that the hexameric compound represents the shortest product of hydrolysis by the enzyme that occurs at a relatively fast rate; the reactivity of hexameric substrates is clearly slow enough to allow crystallisation of the complex. The hexamer was nevertheless recognised by the enzyme as a substrate, since its presence was not detectable in the reaction mixtures after 24 h incubation. Oligomers shorter than six glutamic acid residues did not react or reacted so slowly that they were still present after 30 h reaction. A higher enzyme concentration was required to obtain complete conversion of these oligomers to dimer and trimer. Intriguingly, the hexamer has the right dimension to fit completely across the active site cleft, spanning its whole length. This suggests that we trapped a hexamer because it is the species with sufficiently high affinity for the enzyme, such that the process of crystallisation could compete kinetically against further degradation.

An important aspect of our results is the establishment of the stereoselectivity of this enzyme which showed that the only possible degradation products are L‐glutamates. Accordingly, the peptide trapped in the crystal was clearly an L‐γ‐PGA oligomer.

The structures of PghL and its complex with γ‐PGA thus clarify a number of important issues. They explain why these enzymes are γ‐PGA‐specific carboxypeptidases. We observed that the γ‐PGA substrate is bound to the enzyme through a network of hydrogen bonds with protein side chains. Their spacing is specific for extended γ‐PGA chains but is incompatible with the much shorter spacing of conventional α‐peptides. The structures also suggest how the enzyme achieves stereospecificity. The network of interactions observed with the substrate suggests that stereoselectivity is determined by interaction between the enzyme side chains and L‐γ‐PGA. Some anchoring side chains, such as that of Arg171, are clearly strategically positioned to hold specifically L‐ rather than γ‐D‐PGA explaining the stereospecificity of *B. subtilis* PghL for PGA chains containing L‐glutamate residues 2. Accordingly, we demonstrated that mutation of Arg171 into the shorter serine abolishes completely enzymatic activity. Altogether, this knowledge holds promise for using PghL as a drug design target and for designing mutations in PghL and its homologues that would change the specificity of these enzymes for targeting γ‐D‐PGA‐producing pathogenic bacteria such as *B. anthracis*.

## Materials and methods

### Protein production

Expression of PghL from *B. subtilis* according to the reading frame previously identified 2 was induced from pETSL encoding the *pghL* gene in BL21(DE3) cells (New England Biolabs, Ipswich, MA, USA). A single colony from a freshly transformed *Escherichia coli* plate was used to inoculate 10 mL of 2xYT media supplemented with 100 μg·μL^−1^ of carbenicillin D sodium (Apollo Scientific Limited, Stockport, UK) incubated overnight at 37 °C and agitated at 200 r.p.m. The overnight culture was used to inoculate 1L of 2xYT medium, incubated at 37 °C in a shaking incubator and allowed to grow up to an optical cell density OD_600 nm_ of 0.8. Bacterial cultures were supplemented with 8.33 μm of zinc sulphate just before induction to express the native (zinc‐bound) PghL. Protein expression was initiated by 0.2 mm of (IPTG; Melford Laboratories, Ipswich, MA, USA) at 18 °C overnight. Cells were harvested by centrifugation (Beckman Coulter, Avanti J‐26XP, Brea, CA, USA) at 4 °C. The supernatant was discarded and pellets were suspended in 20 mm Tris/HCl, 300 mm NaCl, at pH 8.0, sonicated (Branson Sonifier 250) and centrifuged (Beckman Coulter, Avanti J‐26XP) at 27 167.4 ***g*** for 45 min. In contrast to previous reports 2 the resulting protein was soluble. The supernatant was loaded onto the gravity column, packed with 5 mL Super Ni‐NTA affinity resin (Generon, Slough, UK), pre‐equilibrated with the resuspension buffer. The His_6_‐tagged apo and native PghL were eluted by using 20 mm Tris/HCl, 300 mm NaCl, 250 mm imidazole pH 8.0. Apo PghL protein fractions were further dialysed against buffer containing 300 mm EDTA. The protein fractions were concentrated to a volume of 3 mL and loaded onto a HiLoad™16/60 Superdex75 prep grade column (GE Healthcare, Chicago, IL, USA) using an AKTA Purifier (Pharma Biotech, Ipswich, MA, USA). The elution profile was monitored by the UV‐absorbance at 280 nm and the purity of the protein was verified by SDS/PAGE gel (NuPage 12% BisTris). The mutant enzyme Arg171Ser was obtained by site‐specific mutagenesis and expressed and purified similar to the wild‐type.

### Protein characterisation

PghL stocks were prepared at a concentration of 5 μm in 20 mm sodium phosphate at pH 6.0, and in 5 mm Hepes (with 150 mm NaF) at pH 6.0. The far‐UV CD spectra were recorded (190–260 nm) on a Jasco J‐715 spectrophotometer using a 1 mm quartz cuvette. For each measurement, multiple scans were accumulated and the baseline was corrected by subtracting the spectrum of the appropriate buffer. Thermal denaturation curves were obtained by monitoring the ellipticity at 222 nm while heating in the range 25 °C–99 °C at a rate of 1 °C·min^−1^. The data were analysed by nonlinear regression analysis assuming a two‐state transition unfolding.

### Chemical reagents and instrumentation for determination of the enzymatic activity

1‐fluoro‐2,4‐dinitrobenzene (Sanger's reagent), Nα‐(2,4‐dinitro‐5‐fluorophenyl)‐L‐valinamide, Dowex 1 × 8 ion exchange resin and HPLC‐grade solvents from Aldrich were used. γ‐PGA (54D:46L) was from Natto Bioscience (Tokyo, Japan). HPLC analyses were carried out on a Jasco LC‐4000 instrument connected to a UV/Vis detector (UV‐4070) and interfaced to a PC running chromnav software package (Jasco, Tokyo, Japan) using a Gemini RP 18 250 × 4.60, 5 μm column (Phenomenex, Torrance, CA, USA).

^1^H NMR spectra were acquired in D_2_O at 400 MHz on a Bruker400 Ultrashield spectrometer. Spectra analyses were carried out with the inmr software (Mestrelab Research, Santiago de Compostela, Spain, www.inmr.net). The ^1^H chemical shifts were:

γ‐GluGlu: δ 4.12 (dd, *J *=* *8.8, 4.6 Hz, 1H), 3.26 (t, *J *=* *6.5 Hz, 1H), 2.34 (t, *J *=* *7.9 Hz, 2H), 2.23 (t, *J *=* *8.1 Hz, 2H), 2.09–2.01 (m, 1H), 1.96–1.77 (m, 3H).

γ‐Glu‐γ‐GluGlu: δ 4.35 (dd, *J *=* *9.1, 5.2 Hz, 1H), 4.29 (dd, *J *=* *9.2, 5.0 Hz, 1H), 3.98 (t, *J *=* *6.5 Hz, 1H), 2.49 (td, *J *=* *7.5, 2.3 Hz, 2H), 2.42 (t, *J *=* *7.3 Hz, 2H), 2.36 (t, *J *=* *7.4 Hz, 2H), 2.18–2.09 (m, 4H), 1.99–1.90 (m, 2H).

### Pre‐column derivatisation with 1‐fluoro‐2,4‐dinitrobenzene (Sanger's reagent)

One hundred microlitre of reaction sample, 400 μL of 0.1 m borate buffer pH 8.5 and 500 μL of 10 mm solution of Sanger's reagent in acetone were mixed in a Pyrex tube equipped with a perforated screw cap and a pierceable septum. The tube was sealed and heated in a water bath at 70 °C in the dark for 45 min. Most of the acetone was evaporated by inserting a needle through the septum and heating for further 10 min. After cooling, equal volumes of the resulting solution and 0.1% aqueous trifluoroacetic acid were mixed and analysed by HPLC using gradient.

### Enzymatic activity

Enzymatic activity PghL is a folded and relatively stable protein. (a) Comparison of the far‐UV spectrum of apo (red) and native PghL (blue) at 30 °C. (b) Thermal scan of PghL monitored by CD. Black curve: PghL in 20 mm sodium phosphate, at pH 6.0 using a protein concentration of 5 μm. The calculated melting point was 55 °C. Blue curve: PghL in 5 mm Hepes and 150 mm NaF at pH 6.0 using a protein concentration of 5 μm. The calculated melting point was 53 °C was followed on agarose gels as previously described 1. Briefly, 100 μg of wild‐type PghL or Arg171Ser mutant were incubated with 4 μL γ‐PGA from *B. subtilis* in 100 mm Tris/HCl pH 8.5 for 1 h at 37 °C, separated on 2% TAE‐agarose and stained with methylene blue.

A more detailed investigation of the activity was carried out by HPLC. After removal of the low‐molecular weight fraction of the commercial material by ultrafiltration using a 10 000 Da cut‐off membrane, the high‐molecular weight γ‐PGA fraction was recovered by lyophilisation. A solution of γ‐PGA in HEPES buffer at pH 7.5 and 0.1 m NaCl was stirred at 37 °C and the reaction was initiated by enzyme addition. Samples were collected, derivatised with Sanger's reagent and analysed by HPLC. After 24‐h digestion, the reaction was checked by HPLC. The pH of the reaction mixture was adjusted to 9.5 with 0.1 m NaOH and loaded on a pad of Dowex 1 × 8 ion exchange resin 200–400 mesh in the acetate form. The pad was eluted with water and increasing concentrations of acetic acid (2–5 m). The eluate was collected in fractions and analysed by TLC. Fractions containing the low‐molecular weight fragments (γ‐GluGlu and γ‐Glu‐γ‐GluGlu) were combined separately and freeze‐dried. Each pool of fractions was redissolved in water (10 mg·mL^−1^) in a Pyrex tube equipped with a screw cap. The tube was sealed after addition of 500 μL of 6 m HCl, and heated at 105 °C in a sand bath for 24 h. The tube was then cooled to room temperature and the mixture was transferred in another Pyrex tube equipped with a perforated screw cap and a pierceable septum. The pH was adjusted to 8.5 with NaOH and 300 μL of 0.1 m borate buffer at pH 8.5 and 450 μL of Nα‐(2,4‐dinitro‐5‐fluorophenyl)‐L‐valinamide 0.15 mg·mL^−1^ solution in acetone were added. After mixing, the tube was sealed and heated at 40 °C in a water bath for 1 h. After evaporation of most of the acetone, the solution was cooled under running water and analysed by HPLC.

### Determination of enzyme stereospecificity

At the end of the reaction the enzyme was inactivated by heating at 90 °C for 20 min. The reaction mixture was then dialysed against water using a membrane with 3500 Da cut‐off. The first portion of water was freeze‐dried affording a mixture of HEPES, γ‐GluGlu and γ‐Glu‐γ‐GluGlu (80 mg), as revealed by ^1^H NMR and HPLC analysis. The high‐molecular weight fraction was recovered from the dialysis tube by freeze drying. The high‐molecular weight fraction was hydrolysed with 6 m HCl and the resulting glutamic acid was analysed by HPLC after pre‐column derivatisation with Nα‐(2,4‐dinitro‐5‐fluorophenyl)‐L‐valinamide.

### Structure determination

Automated crystallisation screening was carried out by sitting‐drop vapour diffusion in 96 well plates (MRC crystallisation plates, Molecular Dimensions), using a Mosquito nanolitre pipetting robot (TTP Labtech, Melbourn, UK) and screens from Molecular Dimensions (Clear Strategy I & II, JCSG‐plus, Morpheus, Structure Screen 1 & 2, Stura Footprint, PACT, Proplex, PGA) and Qiagen (Hilden, Germany) (polyethylene glycols suite, polyethylene glycols II suite, JCSG+ suite, PACT suite). Each crystallisation drop was made by mixing 500 nL of recombinant PghL at a concentration ranging between 14 and 20 mg·mL^−1^ and 500 nL of reservoir solution. PghL crystallised in 4–6 days. Apo PghL and native PghL crystals grew in 1 m ammonium sulphate and 0.15 m sodium citrate (with no pH adjustment). PghL‐γ‐PGA crystals grew in 0.1 m sodium acetate pH 5.0, 5% ^w^/_v_ γ‐PGA, 20% polyethylene glycol 3350. The respective reservoir solutions were used as cryoprotectant prior to flash freezing in liquid nitrogen.

X‐ray diffraction data were collected at the Diamond Light Source (Didcot, UK) for native PghL (1000 frames, 0.1° oscillation) using beamline I03 which was equipped with a Pilatus3 6M detector. Data sets for apo PghL (294 frames, 0.5° oscillation) and PghL‐γ‐PGA complex (314 frames, 0.5° oscillation) were collected in‐house using an Excalibur PX Nova (Oxford Diffraction, United Kingdom). Complete data sets for native PghL, apo PghL and a PghL‐γ‐PGA complex were collected to 1.03, 1.7 and 1.7 Å resolution respectively. All three data sets were collected from one crystal each in the space group P2_1_2_1_2_1_ with similar unit cell dimensions (*a *=* *38.7, *b *=* *47.3, *c *=* *99.9 Å for apo PghL) and one molecule per asymmetric unit. Data were processed using the mosflm software 23, 24 and scaled using the AIMLESS program 25. Initial phases were obtained by the molecular replacement method in PHASER 26 with the coordinates of the model produced in Swiss‐model 27, 28, 29. This model was produced with the coordinates for bacteriophage ΦNIT1 zinc peptidase PghP (PDB accession code: 3a9l). Crystallographic refinement was carried out using PHENIX 30 and REFMAC 31. Model fitting was carried out with the coot v.0.8.2 software 32. The program MOLPROBITY 33 was used to check for structure validations. The detailed statistics for the refined structures of PghL are given in Table 1. The figures were drawn with the pymol graphic program (pymol Molecular Graphics System, v.1.8.0.5, Schrödinger, LLC, New York, NY, USA).

## Conflict of interest

The authors declare no conflict of interest.

## Author contributions

MR produced the protein and carried out protein characterisation, SR crystallised and solved the structure; CC provided the plasmids and promoted the project, CM characterised the protein enzymatically, BJS and AP analysed the results and managed the project. AP wrote the article with contributions from everybody else.
